# Role of Gut Microbiota in Long COVID: Impact on Immune Function and Organ System Health

**Published:** 2025-02-04

**Authors:** Angelie Pathak, Devendra K. Agrawal

**Affiliations:** Department of Translational Research, College of Osteopathic Medicine of the Pacific, Western University of Health Sciences, Pomona, California 91766 USA

**Keywords:** ACE2, Gut dysbiosis, Gut-lung axis, Gut microbiota, Inflammation, Leaky gut, Long COVID, SARS-CoV-2

## Abstract

SARS-CoV-2 infection has led to a range of long-lasting symptoms, collectively referred to as long COVID. Current research highlights the critical role of angiotensin-converting enzyme 2 (ACE2) in regulating gut microbiota diversity, vascular function, and homeostasis within the renin-angiotensin system (RAS). ACE2 is utilized by the SARS-CoV-2 virus to enter host cells, but its downregulation following infection contributes to gut microbiota dysbiosis and RAS disruption. These imbalances have been linked to a range of long COVID symptoms, including joint pain, chest pain, chronic cough, fatigue, brain fog, anxiety, depression, myalgia, peripheral neuropathy, memory difficulties, and impaired attention. This review investigates the dysregulation caused by SARS-CoV-2 infection and the long-term effects it has on various organ systems, including the musculoskeletal, neurological, renal, respiratory, and cardiovascular systems. We explored the bidirectional interactions between the gut microbiota, immune function, and these organ systems, focusing on how microbiota dysregulation contributes to the chronic inflammation and dysfunction observed in long COVID symptoms. Understanding these interactions is key for identifying effective therapeutic strategies and interventional targets aimed at mitigating the impact of long COVID on organ health and improving patient outcomes.

## Introduction

**Figure 1:**
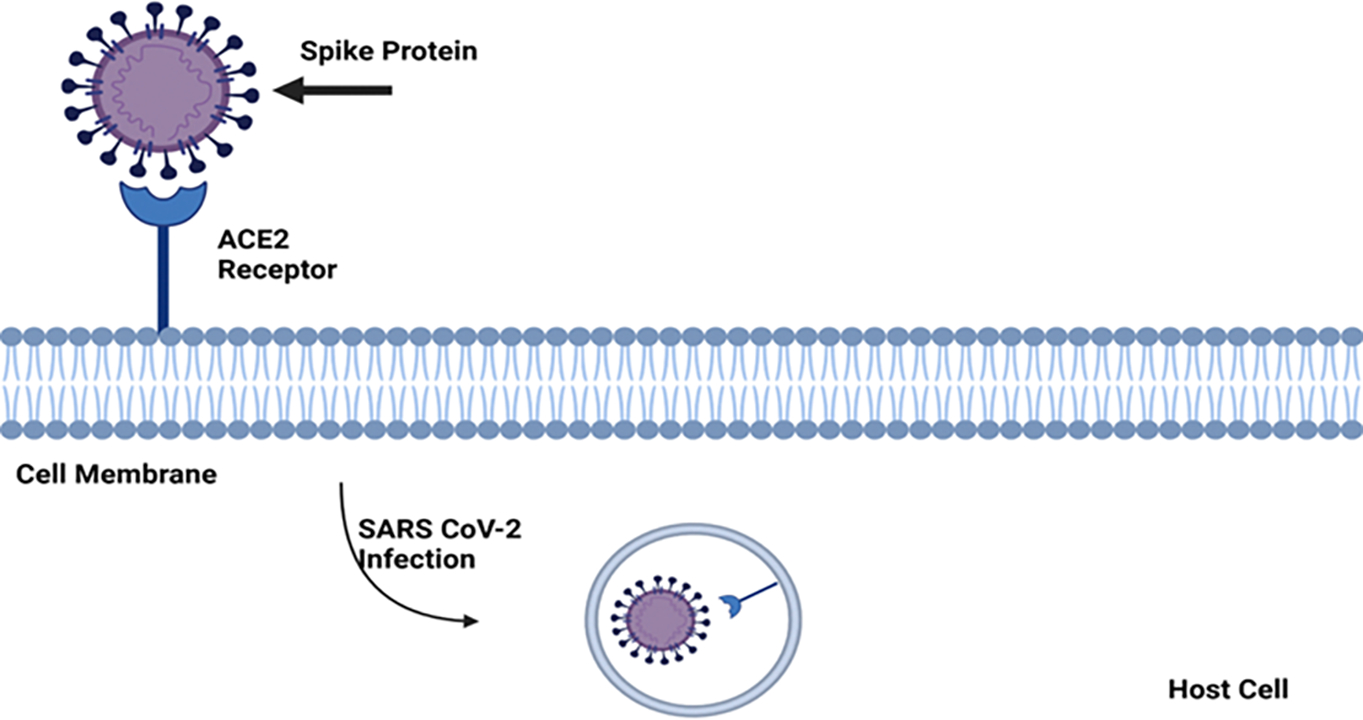
Viral entry of SARS-CoV-2 mediated by the spike protein binding to the ACE2 receptor on the host cell membrane via its receptor-binding domain, which exhibits high affinity for ACE2. Proteolytic cleavage of the spike protein facilitates fusion between the viral envelope and the host cell membrane, enabling the viral genome to enter the host cell.

Severe acute respiratory syndrome coronavirus 2 (SARS-CoV-2) is an airborne virus and the causative agent of COVID-19. SARS-CoV-2 belongs to the Coronaviridae family and the Nidovirales order. A variety of viruses in this order, including Alphacoronavirus and Betacoronavirus, are known to infect mammals and include human pathogens. Conversely, Gamma coronavirus and Delta coronavirus primarily infect avian species and some mammals, with a reduced likelihood of human infection [1, 2]. The structural and genomic features of SARS-CoV-2, including its envelope, spike glycoprotein (S protein), and large single-stranded, positive-sense RNA genome [3], make it especially infectious and adaptable compared to other coronaviruses. The envelope of SARS-CoV-2 is a lipid membrane that surrounds its genetic material, embedding spike glycoproteins within it. This lipid layer provides durability and allows the virus to evade host immune defenses by mimicking host cell structures. The spike proteins enable the virus to bind to angiotensin-converting enzyme 2 (ACE2) receptors on human cells, facilitating fusion of the viral membrane with the host cell membrane (Figure 1). This fusion allows the virus to inject its RNA directly into the host cell, bypassing some of the body’s initial immune defenses [4].

The S protein, essential for cell entry, binds to ACE2 receptors with high affinity, a trait stronger than that of SARS-CoV [5]. A furin cleavage site on the spike protein enables it to be processed by host enzymes, enhancing the virus’s ability to infect cells [6,7]. This efficient binding and entry mechanism contribute to rapid transmission and adaptability of SARS-CoV-2 to various cell types. The RNA genome of the virus is large and positive sense, meaning it can be directly translated by host cell machinery to produce viral proteins immediately upon entry [8]. This efficiency accelerates replication within the host, allowing rapid assembly of new viral particles [9]. The positive-sense RNA genome helps SARS-CoV-2 evade host immune responses by quickly taking control of cellular machinery [10]. Together, these characteristics allow SARS-CoV-2 to effectively enter, replicate, and evade immune defenses, contributing to its widespread transmission.

### Transmission of SARS-CoV-2

SARS-CoV-2 has multiple modes of transmission, including respiratory and aerosol droplets as well as surface contamination [11]. Respiratory droplets travel short distances, ranging from 0–6 feet, typically from actions such as sneezing, coughing, or talking [12]. These droplets are often limited to densely populated areas. In contrast, aerosol droplets can remain airborne for up to 3 hours, presenting a risk in high-density, enclosed spaces. Surface contamination transmission varies and can persist on surfaces for hours to days [13], infecting individuals who touch contaminated areas and then contact their face [14,15].

### Factors Affecting Transmission

There are several factors that may affect the transmission of SARS-CoV-2. The environmental and socio-behavioral characteristics can influence the transmission routes of SARS-CoV-2. These include factors such as ventilation [16], temperature [17], gathering size [18], and mask-wearing [19]. Temperature fluctuations have been correlated with COVID-19 spread. A study of multi-city data showed that a decrease in temperature from 17°C to 7.5°C and a reduction in humidity from 11 g/m^3^ to 6 g/m^3^ were associated with higher COVID-19 incidence [20]. Another study examining COVID-19 spread across 190 countries found temperature to be inversely associated with incidence, with humidity having the strongest correlation at 72%. Stronger wind speeds were linked to lower COVID-19 incidence [21]. These findings highlight seasonal transmission patterns and regions more likely to experience higher COVID-19 and potential long COVID incidence. Ventilation plays a key role in COVID-19 transmission, including both natural and mechanical ventilation [22]. Natural ventilation allows outdoor air to flow indoors without mechanical assistance, typically through windows, doors, or vents. Mechanical ventilation, on the other hand, requires equipment such as fans and duct systems [23]. A study evaluating natural ventilation in schools found that opening windows by 15% with cross-ventilation reduced infection risk to less than 1% when masks were used. In contrast, single-sided ventilation was less effective, with only 30% of the cross-ventilation rate [24]. Further studies revealed that both indoor mechanical and natural ventilation were crucial for reducing spread, particularly in enclosed areas. HEPA filters, outdoor air exchange, and cross-ventilation systems, particularly in high-occupancy spaces, were shown to reduce transmission [25].

Gathering size correlates with COVID-19 spread. A study found that gatherings of 50+ people contributed to 5.4% of transmissions, gatherings of 20+ to 18.9%, and gatherings of 10+ to 25.2% [26]. Further studies investigated 184 group events finding that gatherings of 100 or fewer played a significant role in spread due to higher frequency and lower detection and control rates [27]. Additionally, reducing class size from forty to ten people led to a 30% reduction in transmission [28]. Gather size plays a critical role in reducing gathering size to mitigate spread. Mask use is key in reducing transmission. Effectiveness varies based on layers, fabric, fit, and type of mask [29]. A study across 92 regions found a 19% decrease in the reproduction number with consistent mask use [30]. Mask mandates led to significant decreases in new cases, deaths, and hospital visits within 40 days of enforcement [31]. Surgical masks are most effective in low-viral environments, while N95 masks perform better in high-viral load settings [32].

### COVID-19 Associated Symptoms

**Figure 2:**
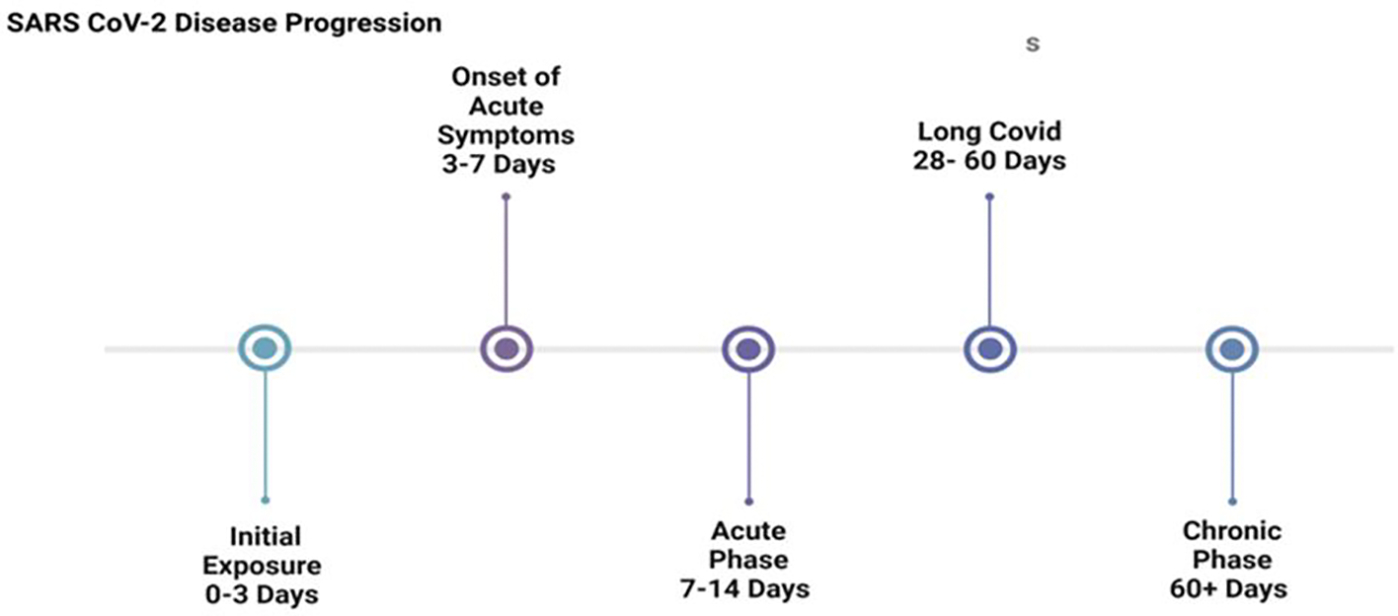
Disease progression of SARS-CoV-2. Days 0–3 typically involve primary and initial symptoms. Between days 3–7, the infection progresses and may intensify, with the viral load peaking at the onset of the acute phase, often accompanied by more severe symptoms. After 14 days, patients may recover or transition to long COVID, characterized by persistent symptoms lasting at least 28 days. Symptoms persisting for over 60 days may lead to the infection being classified as chronic.

SARS-CoV-2 presents a range of symptoms, from asymptomatic to severe outcomes [33]. Symptoms typically appear 2 to 14 days after exposure and may progress in intensity during the infection period. Common symptoms include fever, cough, shortness of breath, headache, body aches, nausea, vomiting, diarrhea, sore throat, congestion, and loss of taste or smell, often lasting 1–2 weeks [34]. In more severe cases, long COVID develops in 28–60 days (Figure 2), characterized by persistent symptoms such as joint pain, chest pain, cough, fatigue, brain fog, anxiety, depression, myalgia, peripheral neuropathy, memory difficulties, and impaired attention. These symptoms can last from 4 weeks to over a year and are linked to factors such as comorbidities, age, occupation, and race-ethnicity [35, 36, 37].

### Comorbidities

Comorbidities are often associated with more severe cases of long COVID [38]. Although a variety of comorbidities can result in worse prognoses, a few have been consistently linked, including hypertension, diabetes, obesity, chronic obstructive pulmonary disease (COPD), asthma, cardiovascular diseases (CVD), liver diseases, malignancy, human immunodeficiency virus (HIV), and renal diseases [39]. These comorbidities have been notably associated with chronic inflammation, immune dysregulation, and metabolic dysfunction, exacerbating viral infections [40]. Obesity and lipid metabolism disorders are the key determinants in the risk for developing long COVID syndrome [41]. In certain cases, comorbidities exhibit more specific symptomatic links. For example, patients with cardiovascular diseases and hypertension demonstrate increased expression of ACE2, which heightens their susceptibility to COVID-19 infection and worsens initial and long-term cardiovascular complications [42, 43]. Similarly, individuals with HIV experience compromised immune responses and heightened inflammation due to decreased CD8+ T cells and increased PD-1+, a key marker for immune exhaustion [44, 45]. Diabetes and obesity also contribute to immune dysregulation by activating pro-inflammatory cytokines and impairing leukocyte metabolism, including reduced T-cell functionality and diminished antibody production [46, 47]. Understanding the various connections between comorbidities and COVID-19 provides valuable insights into predictive methods of infection and highlights the organ systems most likely to be at risk.

### Gut Microbiota

Gut microbiota, comprising protozoa, viruses, fungi, and bacteria, plays essential roles in digestion, metabolism, intestinal barrier support, and immune modulation. Major gut microbiota phyla include Firmicutes, Bacteroidetes, Actinobacteria, and Proteobacteria [48]. These microbiota are influenced by diet, environment, lifestyle, and antibiotic use [49]. Dysbiosis, or imbalance in gut microbiota, is linked to various diseases, including metabolic, gastrointestinal, immune, neurological, psychiatric, and cardiovascular disorders [50]. In the context of SARS-CoV-2, alterations in gut microbiota, such as variations in Ruminococcus gnavus, Bacteroides vulgatus, Faecalibacterium prausnitzii, and Veillonella, have been associated with long-term COVID symptoms, including respiratory dysfunction, fatigue, and chest tightness. Conversely, some microbiota, such as Faecalibacterium prausnitzii and Intestinimonas butyriciproducens, showed protective roles [51, 52].

### Diet and Gut Microbiota

Diet significantly impacts gut microbiota composition and the production of SCFAs (short-chain fatty acids) such as propionate, acetate, and butyrate. Foods with higher levels of resistant starch, inulin, and fructo-oligosaccharides—specifically fruits, vegetables, nuts, and seeds—are positively correlated with gut microbiota producing SCFAs [53, 54]. In contrast, diets high in protein and low in carbohydrates are often associated with decreased SCFA values. Reduced dietary intake of carbohydrates by obese subjects results in decreased concentrations of butyrate and butyrate-producing bacteria in feces [54]. Dietary preferences among global populations differ significantly in these values. Specifically, Mediterranean, plant-based, and vegetarian diets enhance microbial diversity and SCFA production [55, 56]. In an animal study examining a controlled vegetable-protein diet, tight junction integrity increased, thereby reducing pro-inflammatory factors [56]. Additionally, females consuming fermented vegetables presented with increased anti-inflammatory Faecalibacterium prausnitzii but decreased Ruminococcus torques, which typically promote inflammation [57].When comparing various ethnic groups, vegetable intake and red meat consumption were identified as factors influencing microbiota variation. African Americans and Latinos, with lower vegetable intake, exhibited reduced levels of Lachnospira, a microbiota related to fiber breakdown [58]. These findings emphasize the importance in understanding how diet can gut microbiota production and variance to further affect health outcomes and potential prevention or association of disease.

### Race, Ethnicity, Long COVID, and Gut Microbiota

Long COVID and gut microbiota are linked to racial and ethnic variations among populations, exhibiting multidirectional correlations with one another. Studies show that patients with long COVID have reduced bacterial diversity and lower levels of SCFA-producing bacteria even one year after infection [59]. Long COVID disproportionately affects certain racial and ethnic groups. A study in Denmark found that ethnic minorities from North Africa, the Middle East, Asia, and Eastern Europe had a significantly higher risk of infection compared to the native Danish population. Among these groups, individuals of Iraqi, Turkish, and Somali origin had the highest adjusted hazard ratios [60]. Similarly, in the U.S., the Census Bureau’s Household Pulse Survey reported that long COVID was more prevalent among females, Black individuals, and Hispanics compared to males, non-Hispanic individuals, and Whites [61]. These findings align with another U.S.-based study across 12 states, which found that Black individuals had the highest levels of virus-associated hospitalizations. Additionally, Hispanic, Alaska Native, and Native American populations showed a high prevalence of hospitalization [62, 63].

Gut microbiota composition with functional importance in gut integrity and immune functioning varies across racial and ethnic groups. Studies have noted a significant impact of microbiota composition and disease outcomes of COVID-19 [64]. Particularly the Christensenellaceae family, with strong anti-inflammatory roles, has seen a protective role in Long Covid, with Dutch populations experiencing highest levels [65, 66]. A U.S.-based study of students revealed that Black students had higher rates of Bacteroides, producing toxin increasing inflammation, while White students exhibited higher levels of anti-inflammatory microbiota such as Faecalibacterium and Roseburia [67, 68, 69]. Studies on East Asian communities noted more gut microbiota producing SCFAs, which were not directly correlated with diet [70]. Another study found individuals of Ghanaian heritage to have greater microbial diversity and SCFA production, while Americans exhibited the lowest levels [71]. These disparities and variations in long COVID prevalence and gut microbiota may arise from a combination of factors, including socioeconomic inequalities, healthcare access, occupation, evolutionary mechanisms, genetics, housing conditions, and dietary access. The influence of gut microbiota on long COVID outcomes is significant: anti-inflammatory microbiota may help reduce the severity of viral infections and inflammatory responses, while pro-inflammatory microbiota can exacerbate viral symptoms. These differences may partially explain the varying impacts of long COVID across racial and ethnic groups.

### ACE2 Enzyme and Its Role in Gut Microbiota and Long COVID

**Figure 3:**
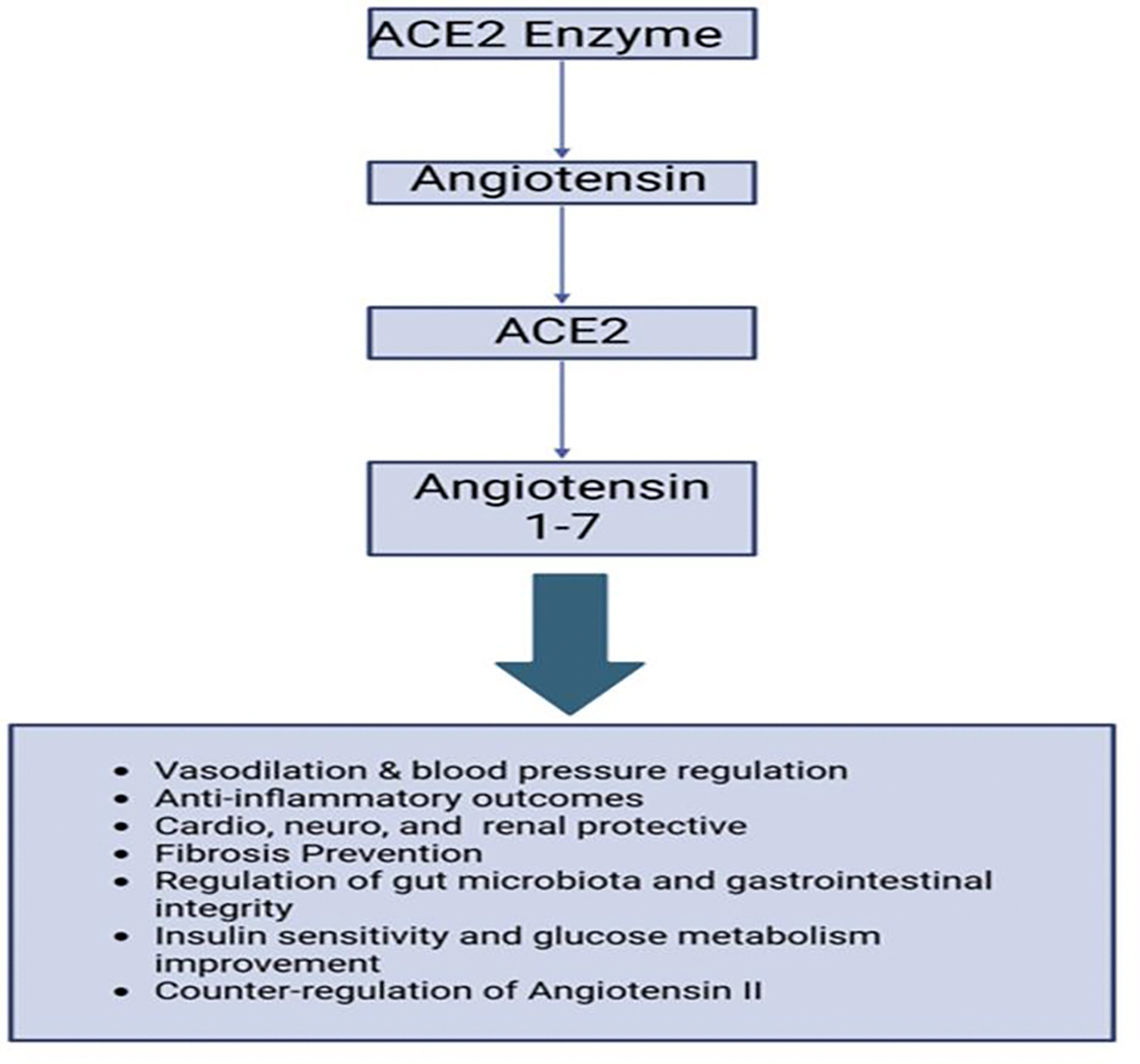
Angiotensin-converting enzyme (ACE) converts angiotensin I to angiotensin II through the process of cleavage. ACE2, a homologue of ACE, converts angiotensin II to angiotensin 1–7 by removing an amino acid. ACE promotes vasoconstriction, while ACE2 acts as a vasodilator.

Angiotensin-converting enzyme (ACE) converts angiotensin I to angiotensin II through the process of cleavage. ACE2, a homologue of ACE, converts angiotensin II to angiotensin 1–7 by removing an amino acid. ACE promotes vasoconstriction, while ACE2 acts as a vasodilator (Figure 3). The ACE2 enzyme plays a crucial role in regulating gut microbiota and is tightly linked to the renin-angiotensin system (RAS), which controls blood pressure, electrolyte balance, and fluid levels [72]. Studies have shown that ACE2 influences gut microbiota composition. In mice, ACE2 knockout leads to higher levels of certain gut microbiota, while overexpression of ACE2 results in increased levels of anti-inflammatory probiotics and SCFA-producing microbiota [73]. This suggests a bidirectional relationship between ACE2 and gut microbiota, where changes in one can influence the other. ACE2 is expressed in both the gastrointestinal and respiratory tracts, making it a key player in the pathogenesis of COVID-19, including long COVID. SARS-CoV-2 binds to ACE2 receptors, downregulating ACE2 expression, which disrupts RAS and gut microbiota homeostasis. This dysregulation contributes to the severity of respiratory symptoms, including chronic cough, shortness of breath, and chest pain in long COVID patients [74, 75]. Furthermore, ACE2 is involved in the kallikrein-kinin system (KKS), which regulates bradykinin levels. When ACE2 is downregulated by SARS-CoV-2 binding, KKS becomes dysregulated, leading to inflammation, vascular permeability, and lung damage, further exacerbating respiratory symptoms [76, 77,78]. In normal functioning, ACE2 converts angiotensin II into angiotensin 1–7, which has vasodilatory and anti-inflammatory effects. However, when ACE2 function is reduced due to SARS-CoV-2 infection, angiotensin II levels remain elevated while angiotensin 1–7 is not produced, contributing to the pathogenesis of cytokine storms in severe COVID-19 cases [79]. These cytokine storms result from an overactive immune response characterized by elevated levels of inflammatory cytokines, which contribute to systemic damage and severe respiratory issues [80].

### Gut Axis and Dysbiosis in Long COVID

ACE2 plays a vital role in maintaining homeostasis in the gut microbiota. If ACE2 expression is reduced, it leads to gut dysbiosis, an imbalance in the microbiota that can result in systemic inflammation, further exacerbating the severity of COVID-19 [81]. This is particularly important in Long COVID, where dysbiosis in the gut microbiota is linked to chronic respiratory symptoms and inflammatory processes.

**Figure 4:**
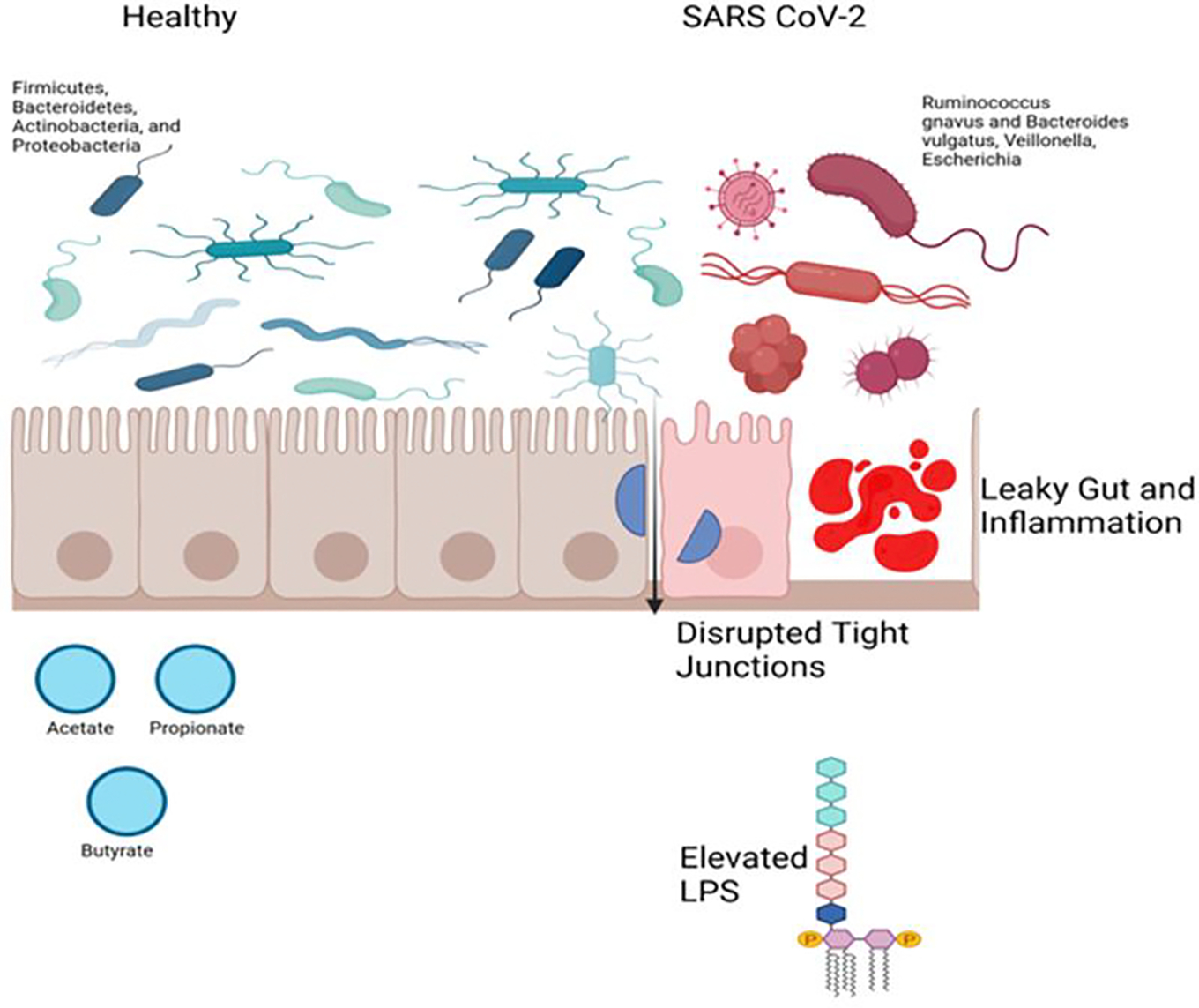
Comparison of the gastrointestinal epithelium in healthy and SARS-CoV-2. In SARS-CoV-2 infection, there is increased levels of lipopolysaccharides (LPS) due to bacterial infection that disrupts the epithelial tight junction, resulting in leaky gut and inflammation.

The gut-lung axis, a bidirectional communication pathway between the gastrointestinal and respiratory systems, plays a key role in this relationship. Disruptions in the gut microbiota affect the immune system, which in turn influences the lungs, leading to chronic inflammation. The dysregulation of ACE2 and the associated gut microbiota imbalance contribute to this process by increasing intestinal permeability. When ACE2 is downregulated, intestinal junction proteins like occludin, claudin, and ZO-1 are compromised, allowing bacterial endotoxins such as lipopolysaccharide (LPS) to leak into systemic circulation (Figure 4). These endotoxins bind to TLR4 receptors on immune cells, triggering the release of pro-inflammatory cytokines like IL-6, TNF-alpha, and IL-1β, which further fuel the cytokine storm and increase inflammation throughout the body [82].

### Gut Dysbiosis and Affected Organ systems in Long COVID

**Figure 5:**
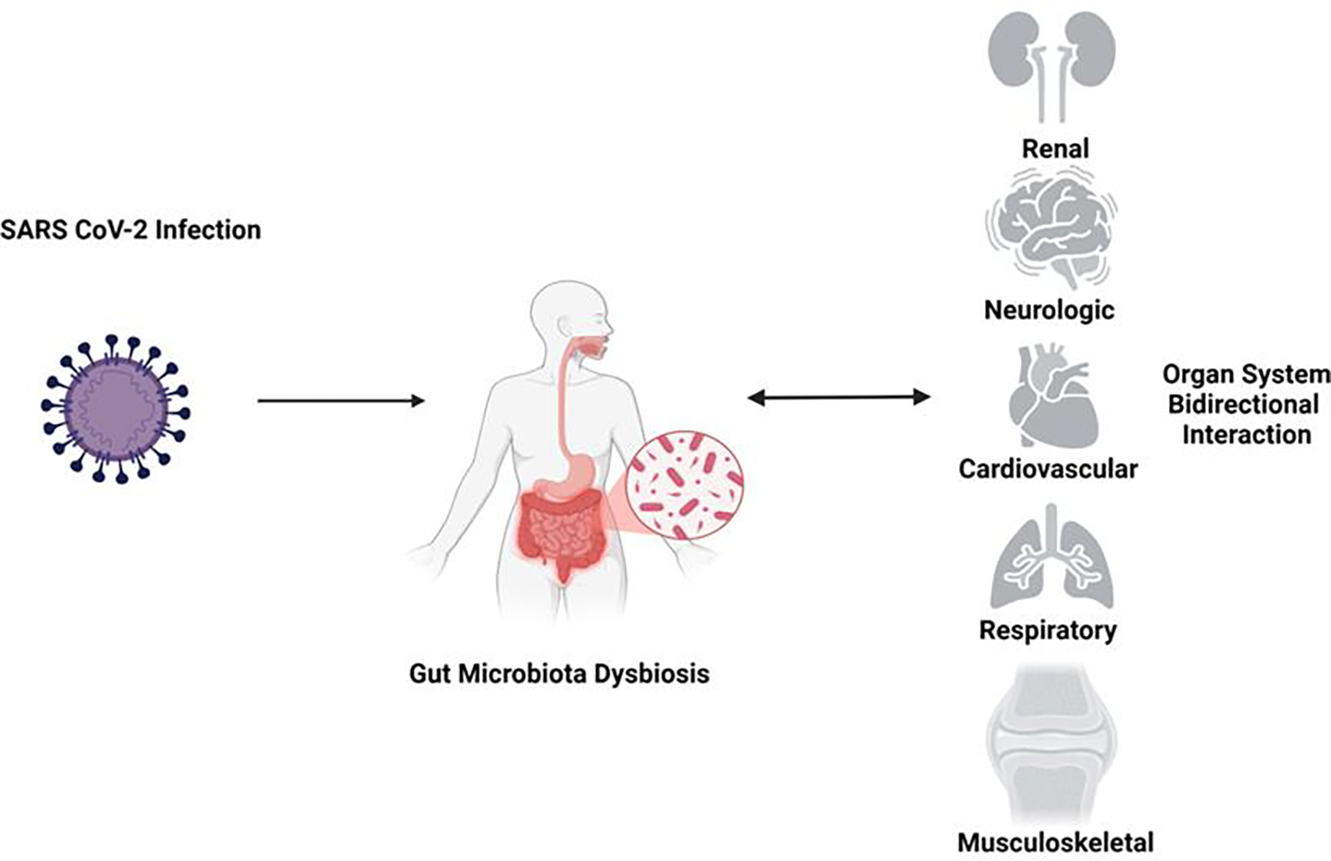
Gut dysbiosis induced by viral infection and its bidirectional effects in relation to long COVID, impacting various organ systems. The most affected systems include the renal, cardiovascular, respiratory, and musculoskeletal systems.

Various organ systems are affected due to gut dysbiosis in long COVID. The most affected systems include the renal, cardiovascular, respiratory, and musculoskeletal systems (Figure 5). Each of the affected systems are discussed below.

### Musculoskeletal System

Recent research has highlighted a significant association between gut microbiota dysbiosis and musculoskeletal (MSK) pain in individuals suffering from Long COVID. Dysbiosis has been shown to influence nocioplastic pain, a form of chronic pain characterized by tissue damage with an unclassifiable origin [83]. Pain in this context is linked to proinflammatory cytokine release. Patients with long COVID experiencing MSK pain exhibit elevated levels of proinflammatory cytokines, which may contribute to the onset and persistence of pain symptoms [84]. One mechanism through which dysbiosis may affect bone metabolism and MSK health involves Toll-like receptor 5 (TLR5). The microbiota acts as an activator of TLR5, which modulates immune responses and impacts bone remodeling. In a healthy system, TLR5 activation by microbiota-derived signals influences the balance between osteoclasts and osteoblasts, both critical for bone homeostasis [85]. Studies on TLR5-deficient mice revealed increased periosteal bone formation, suggesting a potential connection between TLR5 signaling, the microbiome, and bone remodeling [86]. These findings underscore the indirect role of microbiota in bone health and MSK pain via immune modulation, especially when immune balance is disrupted by dysbiosis. Physical activity plays a crucial role in mitigating these effects. Low physical activity, combined with dysbiosis, can exacerbate bone degeneration and MSK pain. Exercise is known to influence gut microbiota, potentially alleviating some of the adverse effects of dysbiosis on the musculoskeletal system [87, 88]. Long COVID patients often report pain in regions such as the knees, shoulders, and cervical spine, with IL-6, IL-10, TNF-α, and IFN-g identified as predictive cytokines for pain in these areas [84]. These cytokines, elevated in individuals with chronic MSK pain, contribute to inflammation and pain sensitivity. Further research has shown that gut microbiota composition influences conditions like lower back pain (LBP) [89]. Higher levels of Adlercreutzia bacteria were associated with increased inflammation and a greater likelihood of developing LBP, particularly in individuals with higher BMI [90]. This highlights the role of specific microbial communities, combined with obesity, in the severity of MSK pain. The presence of pathogenic microbiota, such as Pseudomonas veronii, Pseudomonas stutzeri, and Streptococcus anginosus, has been linked to intervertebral disk degeneration [91]. Dysbiosis-related infections and the subsequent increase in proinflammatory cytokines may directly compromise the structural integrity of the spine and other joints, contributing to chronic pain [92]. Moreover, gut microbiota dysbiosis, when combined with low physical activity, depression, and anxiety, significantly contributes to chronic MSK pain in long COVID patients. The interplay between microbial imbalances, immune system activation, and cytokine release underlies the mechanisms of pain in these individuals [93].

### Neurologic system

Long COVID is increasingly associated with persistent neurological symptoms and cognitive dysfunction, even in non-hospitalized individuals. Common symptoms include brain fog, headaches, numbness or tingling, dysgeusia (altered taste), anosmia (loss of smell), and myalgias (muscle pain) [94]. Among these, brain fog is particularly notable for its strong correlation with long-term cognitive deficits, such as problems with attention, memory, processing speed, and executive function [95]. ACE2 is present in regions without a blood-brain barrier, such as the hypothalamus and circumventricular organs, as well as in regions with a blood-brain barrier, specifically within endothelial cells, astrocytes, and pericytes. The conversion of angiotensin II to angiotensin 1–7 by ACE2 reduces oxidative stress and inflammation at the blood-brain barrier. However, COVID-19-associated downregulation of ACE2 can increase vulnerability to SARS-CoV-2 by promoting viral entry in regions lacking the barrier and compromising blood-brain barrier integrity in protected regions [96, 97]. The olfactory bulb serves as a key entry point for the virus, allowing it to spread to other brain regions, including the brainstem, which has a high density of ACE2 receptors. The brainstem controls essential functions like breathing and heart rate but also plays a role in neurocognitive processes. Its involvement can lead to long-term neurological effects [98].

The causes of brain fog in long COVID are multifactorial, involving mechanisms such as nerve infection, blood-brain barrier disruption and permeability, and inflammation mediated through ACE2 receptors. The virus may enter the brain via several pathways, including the olfactory, trigeminal, and vagus nerves, which have direct connections to the central nervous system and significantly impact neurocognitive functioning [99, 100]. Additionally, the blood-brain barrier, which protects the brain from harmful substances, is thought to be compromised during COVID-19 infection. This disruption, involving the breakdown of tight junctions and dysfunction of endothelial cells and pericytes, allows entry of pathogens and inflammatory molecules, contributing to neurological deficits [101]. Animal studies suggest that elevated levels of angiotensin II can amplify brain inflammation, increasing BBB permeability and neurocognitive symptoms like brain fog [102]. ACE2 receptors located on astrocytes and neurons allow viral binding, which activates brain mast cells, microglia, and astrocytes. This activation releases pro-inflammatory cytokines, impairing brain function and contributing to brain fog [103]. The gut-brain axis is a critical link between the gut microbiome and brain function. ACE2 receptors are present in both the brain and gut, indicating a potential connection between gut health and neurocognitive symptoms. In long COVID, gut microbiota dysbiosis may reduce the production of SCFAs such as butyrate, which is essential for maintaining gut barrier integrity and controlling inflammation. A deficiency in butyrate weakens the gut barrier, contributing to systemic inflammation that can impact brain function. Furthermore, the gut microbiota regulates anti-inflammatory cytokines, helping control systemic inflammation. Dysregulation of this system can lead to a pro-inflammatory state that affects the brain and contributes to neurocognitive symptoms, such as brain fog [104].

### Renal System

Human and mouse model studies have highlighted significant negative effects, and a high prevalence of acute kidney disease (AKD) associated with long COVID [105]. Patients with AKD face a threefold increase in mortality risk. In hospitalized COVID-19 patients, damage to proximal kidney tubules has been observed through urine and biochemical marker tests. Proximal kidney tubules with ACE2 receptors facilitate viral binding. Combined with cathepsins that modify spike proteins, these receptors allow the virus to enter through the apical membrane, leading to renal dysfunction [106]. Severe COVID-19 and prolonged long COVID increase the risk of acute kidney injury (AKI), often resulting in proteinuria. Patients with pre-existing chronic kidney disease (CKD) and long COVID have experienced reductions in glomerular filtration rates (GFR), with a median decrease of 2.96 mL/min/1.73 m^2^ in virus-infected patients [107]. Studies also report that a GFR <60 mL/min/1.73 m^2^ correlates with higher hospital mortality rates in long COVID patients [108]. Additionally, long COVID patients are at greater risk of developing end-stage kidney disease (ESKD), defined as a GFR <15 mL/min/1.73m^2^, leading to kidney failure that requires dialysis or a transplant for proper body regulation [109]. Postmortem renal histopathological analysis of COVID-19 patients in China has revealed significant kidney tissue damage, including dilation, thinning, and flattening of the tubules [110]. This damage is primarily attributed to viral infection, which disrupts kidney function, particularly in the proximal tubular cells (PTCs) that are essential for nutrient and electrolyte reabsorption [111]. Dysregulation of the gut microbiota in long COVID patients is linked to the accumulation of uremic toxins such as indoxyl sulfate, p-cresyl sulfate, and trimethylamine N-oxide. These toxins contribute to chronic inflammation and endothelial dysfunction [112]. Specifically, they disrupt junctional proteins like VE-cadherin and ZO-1, leading to increased endothelial permeability and vascular inflammation, which further exacerbates kidney and systemic dysfunction [113]. Histological findings in long COVID patients have shown similar renal pathology [114]. COVID-induced kidney injury impairs the primary function of PTCs, disrupting the reabsorption of essential nutrients, electrolytes, minerals, and amino acids, particularly tryptophan. Tryptophan is crucial for the tryptophan–kynurenine pathway and the production of melatonin and serotonin. Deficiencies in tryptophan can affect neurocognitive functioning, contributing to brain fog, fatigue, and muscle weakness—symptoms commonly associated with long COVID [115].

### Respiratory System

ACE2 is expressed on type II alveolar epithelial cells within the lungs, where it facilitates gas exchange and the production of surfactant. Surfactant is essential for maintaining alveolar stability, allowing efficient oxygen and carbon dioxide exchange between the bloodstream and air. ACE2 also plays a critical role in the renin-angiotensin system (RAS), modulating inflammation, angiotensin II-mediated vasoconstriction, and vascular permeability [116, 117]. The mucosal immune system forms the primary defense against pathogens across mucosal surfaces, including the respiratory tract, gastrointestinal tract (GIT), and genitourinary tract. Components of this system include epithelial cells, mucosal layers, gut-associated lymphoid tissue (GALT), nasopharyngeal-associated lymphoid tissue (NALT), immune cells, secretory IgA, cytokines, and chemokines. The interconnectedness of immune responses across mucosal sites, referred to as the common mucosal immune system (CMIS), is evidenced by the migration of B immunoblasts into intestinal, respiratory, and genital tissues, highlighting a shared immune response [118]. The gut microbiota significantly regulates mucosal immune function, influencing the development of immune cells such as Th17 and regulatory T cells. These interactions are mediated through microbiota-derived metabolites, such as SCFAs, which support epithelial barrier integrity and modulate immune cell activity. The gut microbiota is also essential for maintaining immune tolerance, preventing inappropriate inflammation [119]. The gut-lung axis exemplifies the interconnected nature of mucosal immunity. Studies show that respiratory infections can influence gastrointestinal immunity and vice versa. For example, respiratory influenza virus infections have been linked to intestinal immune injury mediated by microbiota-driven Th17 cell inflammation [120]. Similarly, disruptions in the gut microbiota can affect respiratory immunity, demonstrating the bidirectional communication within this axis.

In long COVID, dysbiosis in the gut microbiota is increasingly implicated in chronic respiratory symptoms. Markers such as lipopolysaccharide-binding protein (LBP), which indicate gut microbiota dysfunction, have been closely associated with respiratory failure in COVID-19 patients [121]. These findings underscore the role of gut-lung axis disruptions in exacerbating respiratory dysfunction. Persistent respiratory complications in long COVID, including chronic cough, fibrotic lung disease, bronchitis, and pulmonary vascular disease, are frequently linked to chronic inflammation [122]. Dysbiosis in the gut microbiota, characterized by reduced microbial diversity, may contribute to this inflammation, affecting both the GIT and respiratory systems. These findings highlight the need to explore the microbiota’s role in immune regulation as a pathway for developing therapeutic strategies for managing long COVID.

### Cardiovascular System

The pandemic presented patients afflicted with SARS-CoV-2 with a range of cardiovascular complications, including myocarditis, stress cardiomyopathy, myocardial infarction (MI), and arrhythmias [123]. One study found that 80% of patients with severe COVID-19 experienced some level of cardiac symptoms, and 25% reported persistent symptoms three months post-diagnosis [124]. A variety of potential mechanisms could contribute to these outcomes, with bidirectional interactions between comorbidities and viral infection impacting cardiac function [125]. A systematic review by Sha’ari et al. revealed that COVID-19-infected patients with pre-existing CVD had significantly higher risks of long COVID, with hypertension and heart failure serving as the strongest predictors [126]. Additional studies corroborate these findings, linking hypertension, elevated cholesterol levels, and CVD comorbidities to post-viral chronic symptoms, further emphasizing the multidirectional relationship among the cardiovascular system, gut dysbiosis, and long COVID [127]. The ACE2 receptor, expressed on cardiac pericytes, plays a role in regulating myocardial blood supply. SARS-CoV-2 infection can impair ACE2 function, causing capillary endothelial dysfunction and restricted myocardial blood flow, potentially contributing to cardiovascular complications [128]. Endothelial cells, which also express ACE2, are susceptible to acute vasculitis, a possible mechanism of cardiovascular injury.

SCFAs act on endothelial cells to prevent vascular cell adhesion molecule-1 (VCAM-1) and IL-6 and IL-8 pro-inflammatory expression, reducing inflammation and cell adhesion to prevent atherosclerosis [129]. SCFAs further regulate blood pressure by reducing oxidative stress and modulating neurohormonal pathways, mitigating negative cardiovascular effects [130]. Additionally, SCFAs modulate lipid metabolism, decreasing triglyceride, cholesterol, and LDL levels while increasing HDL levels [130]. Gut microbiota, characterized by decreased diversity, low SCFA levels, and systemic inflammation, has emerged as a key contributor to cardiovascular outcomes in SARS-CoV-2 infection [131, 132]. The gut microbial metabolite trimethylamine N-oxide (TMAO), derived from microbial metabolism of dietary choline, L-carnitine, and betaine, is implicated in cardiovascular disease through mechanisms such as inhibition of cholesterol metabolism, arterial plaque formation, platelet aggregation, and thrombosis [133]. TMA-producing bacteria in the order Clostridiales are instrumental in TMAO production [134]. COVID-19 infections can exacerbate gut dysbiosis, altering the Clostridiales order, including decreased levels of anti-inflammatory species such as Faecalibacterium prausnitzii and increased pathogenic species such as Clostridium ramosum and Clostridium hathewayi [135, 136]

Faecalibacterium prausnitzii plays a role in SCFA butyrate production, strengthening gut wall integrity. Higher levels of this bacterium are associated with reduced coronary heart disease and ischemic stroke. It has also been linked to reduce inflammation by decreasing plasma LPS levels [137, 138]. Global studies have further strengthened the connection between long COVID, gut dysbiosis, and cardiovascular outcomes. One study, conducted across 12 countries, found that patients more likely to be hospitalized with COVID-19 exhibited lower levels of Faecalibacterium prausnitzii and higher levels of pro-inflammatory bacteria, exacerbating cardiovascular issues [139]. Another study, involving 2,871 adult subjects from 16 countries, identified a significant association between low levels of butyrate-producing bacteria and COVID-19 mortality [140]. These findings confirm the multifaceted connection between gut microbiota, the cardiovascular system, and the symptoms experienced by long COVID patients, highlighting the value of integrative approaches in preventing cardiovascular symptoms and improving long-term outcomes.

### Summary

The role of gut microbiota in long COVID symptoms remains to be fully understood. However, current research highlights the significant impact of gut dysbiosis on both health and disease states across various organ systems. Organ-specific comorbidities may bidirectionally exacerbate gut dysbiosis and Long COVID symptoms. Notably, dysregulation involving the ACE2 receptor, SCFA production levels, cytokine storms, and pro-inflammatory responses has been identified as central to this process. Further research is needed to delineate the specific systems directly versus indirectly affected, as well as to understand the cross-communicative interactions between systems.

### Future Directions

Accounting for geographical, environmental, socio-behavioral, dietary, comorbidity, and racial-ethnic disparities is essential for understanding the observed outcomes of gut dysbiosis associated with Long COVID. Future studies should prioritize longitudinal tracking of gut microbiota from the pre-infection stage through acute infection and into the chronic phase of Long COVID. More targeted approaches should include person-specific microbiome profiling, particularly for individuals with comorbidities who are at higher risk of developing long COVID. These studies should examine microbiome shifts over time during infection and explore interventions tailored to these high-risk patients. Moreover, efforts should focus on monitoring individuals at elevated risk before infection to evaluate how probiotics and targeted gut therapies might reverse or mitigate gut inflammation and dysbiosis. Such interventions could help maintain organ-specific balance and reduce chronic symptoms. This research should also consider controlled dietary and environmental factors to explore how supplementation and fortification could act as preventive mechanisms against Long COVID. Using the microbiome as a predictive tool holds promise for improving primary preventive outcomes. On a global scale, controlled supplementation studies within diverse populations—considering diet, environment, race, and ethnicity—could identify which groups benefit most from specific interventions and which may require multidisciplinary preventive approaches. Understanding the influence of microbiome shifts on the progression of Long COVID could uncover early markers of the disease. This would help clarify the predictive value of the changes in the microbiota and play a central role in symptom mitigation, ultimately improving long-term health outcomes for patients.

## Key Findings

- SARS-CoV-2 infection and transmission are influenced by factors such as ventilation types, mask usage, and gathering size. Additionally, race and ethnicity, along with variations in gut microbiota composition, contribute to differences in susceptibility and disease severity.
- Pre-existing conditions may increase the risk of severe COVID-19 and long COVID. These conditions can contribute to chronic inflammation and immune dysfunction, exacerbating symptoms.
- Dysbiosis in the gut microbiota, influenced by ACE2 downregulation, contributes to systemic inflammation, exacerbating chronic respiratory symptoms and inflammatory processes in Long COVID.
- Disruption of the gut-lung axis, through immune system modulation and bacterial endotoxins like LPS, increases chronic inflammation, worsening respiratory and musculoskeletal symptoms in Long COVID patients.
- Long COVID has bidirectional effects on gut microbiota and multiple organ systems, including musculoskeletal, renal, neurological, cardiovascular, and respiratory systems. Gut microbiota dysbiosis influences the severity and progression of long COVID symptoms across these organ systems.
